# Molecular characterization of a new IgZ3 subclass in common carp (*Cyprinus carpio*) and comparative expression analysis of IgH transcripts during larvae development

**DOI:** 10.1186/s12917-021-02844-7

**Published:** 2021-04-14

**Authors:** Fumiao Zhang, Mojin Li, Cui Lv, Guangcai Wei, Chang Wang, Yimeng Wang, Liguo An, Guiwen Yang

**Affiliations:** 1 Key Laboratory of Animal Resistance Biology of Shandong Province, College of Life Sciences, Shandong Normal University, No. 88 East Wenhua Road, Shandong 250014 Jinan, China; 2 Laboratory of Immunology for Environment and Health, Shandong Analysis and Test Center, Qilu University of Technology (Shandong Academy of Sciences), 250014 Jinan, China

**Keywords:** Common carp, Immunoglobulin, IgZ, Ontogeny

## Abstract

**Background:**

Immunoglobulins (Igs) distributed among systemic immune tissues and mucosal immune tissues play important roles in protecting teleosts from infections in the pathogen-rich aquatic environment. Teleost IgZ/IgT subclasses with different tissue expression patterns may have different immune functions.

**Results:**

In the present study, a novel secreted IgZ heavy chain gene was cloned and characterized in common carp (*Cyprinus carpio*). This gene exhibited a different tissue-specific expression profile than the reported genes IgZ1 and IgZ2. The obtained IgZ-like subclass gene designated *Cc*IgZ3, had a complete open reading frame contained 1650 bp encoding a protein of 549 amino acid residues. Phylogenetic analysis revealed that *Cc*IgZ3 was grouped with carp IgZ2 and was in the same branch as IgZ/IgT genes of other teleosts. Basal expression detection of the immunoglobulin heavy chain (IgH) in healthy adult common carp showed that *Cc*IgZ3 transcripts were widely expressed in systemic immune tissues and mucosal-associated lymphoid tissues. *Cc*IgZ3 was expressed at the highest levels in the head kidneys, gills, and gonads, followed by the spleen, hindgut, oral epithelium, liver, brain, muscle, foregut, and blood; it was expressed at a very low level in the skin. The transcript expression of *Cc*IgZ3 in leukocytes isolated from peripheral blood cells was significantly higher than that in leukocytes isolated from the spleen. Different groups of common carp were infected with *Aeromonas hydrophila* via intraperitoneal injection or immersion. RT-qPCR analysis demonstrated that significant differences in *Cc*IgZ3 mRNA levels existed between the immersion and injection groups in all the examined tissues, including the head kidney, spleen, liver, and hindgut; in particular, the *Cc*IgZ3 mRNA level in the hindgut was higher in the immersion group than in the injection group. The different routes of *A. hydrophila* exposure in common carp had milder effects on the IgM response than on the *Cc*IgZ3 response. Further study of the relative expression of the IgH gene during the development of common carp showed that the tissue-specific expression profile of *Cc*IgZ3 was very different from those of other genes. RT-qPCR analysis demonstrated that the *Cc*IgZ3 mRNA level increased gradually in common carp during the early larval development stage from 1 day post fertilization (dpf) to 31 dpf with a dynamic tendency similar to those of IgZ1 and IgZ2, and IgM was the dominant Ig with obviously elevated abundance. Analyses of the tissue-specific expression of IgHs in common carp at 65 dpf showed that *Cc*IgZ3 was expressed at mucosal sites, including both the hindgut and gill; in contrast, IgZ1 was preferentially expressed in the hindgut, and IgZ2 was preferentially expressed in the gill. In addition to RT-qPCR analysis, *in situ* hybridization was performed to detect *Cc*IgZ3-expressing cells and IgM-expressing cells. The results showed that *Cc*IgZ3 and IgM transcripts were detectable in the spleens, gills, and hindguts of common carp at 65 dpf.

**Conclusions:**

These results reveal that *Cc*IgZ3 gene transcripts are expressed in common carp during developmental stage not only in systemic tissues but also in mucosal tissues. *Cc*IgZ3 expression can be induced in immune tissues by *A. hydrophila* challenge via immersion and intraperitoneal injection with significantly different expression profiles, which indicates that *Cc*IgZ3 is involved in the antimicrobial immune response and might play an important role in gut mucosal immunity.

## Background

Fish, like other vertebrates, possess an extensive defence system that enables each individual to survive and maintain its integrity in a hostile environment. The humoral immune system responds to a variety of pathogens by producing specific antibodies. Antibodies produced by B lymphocytes exist in vertebrates from mammals to cartilaginous fish [1]. There are five types of heavy (H) chains in mammals, including µ, δ, γ, ε, and ɑ, which differ in their constant regions. The antibodies that contain these different H chains are considered different isotypes and are named immunoglobulin (Ig) M, IgD, IgG, IgE, and IgA, respectively. Each isotype has distinct physical and biological properties and effector functions [2]. Fish are the most primitive group of vertebrates that possess an adaptive immune system capable of generating antibodies in response to pathogenic challenges. Aside from IgM and IgD, a novel isotype, IgZ (ζ) or IgT (τ), has been identified in many teleost fish species [3], This isotype has also been found to have varied subclasses with different gene sequences and different tissue expression patterns, such as IgZ1 and IgZ2 in zebrafish (*Danio rerio*) [4, 5]; IgT1, IgT2, IgT3, IgT4, and IgT5 in rainbow trout (*Oncorhynchus mykiss*) [6]; IgZ and a chimeric IgZ (IgZ2) in grass carp (*Ctenopharyngoden idella*) [7]; IgT1, IgT2, IgT3 and IgT4 in stickleback (*Gasterosteus aculeatus*) [8]; five IgT-A and three IgT-B in Atlantic salmon (*Salmo salar*) [9]; and IgZ1 and IgM-IgZ (IgZ2) in common carp (*C. carpio*) [10]. Most of the IgZ/T molecules have four CH domains that are encoded by the Cζ/Cτ genes, but others have two CH domains or three CH domains; for example, there are two constant domains in common carp IgZ2 [11] and torafugu (*Takifugu rubripes*) IgH [12] and three constant domains in stickleback IgT [8] and sea bass (*Dicentrarchus labrax*) IgT [13]. In addition to exhibiting diversity in domain numbers, the IgZ/T subclasses display varied expression patterns and functions among fish species. The reported IgZ1 of common carp is expressed mainly in blood and has activity against blood pathogens, while the IgZ2 chimera is preferentially expressed in the mucosal compartment to respond to mucosal infections [10]. The rainbow trout Igτ1 is expressed mainly in both systemic and mucosal lymphoid tissues, while Igτ2 is expressed largely in systemic lymphoid organs. After poly (I:C) treatment, the Igτ1 and Igτ2 genes exhibit different expression profiles, and Igτ1 transcript levels peak at 7 days in the spleen and 14 days in the gut. However, Igτ2 levels increase slightly, peaking at 7 days in both the spleen and gut [6]. Thus, previous research on this novel immunoglobulin heavy chain has indicated that teleost fish IgZ/T is more diverse than previously thought.

The teleost IgZ/T considered a primitive Ig class specialized in mucosal immunity, is equivalent to the IgA in mammals and plays an important role in the mucosal immune response [3, 14]. IgZ/T is a comparatively new teleost Ig class, and its presence, expression, and tissue distribution during the early developmental stage are not very clear. Many studies have investigated the ontogeny of IgM-positive cells and IgM-secreting cells. Previous research has indicated that the first B cells are most likely generated in the head kidneys and that B cells populate gut-associated lymphoid tissue (GALT) much later than the spleen or kidneys [15]. In channel catfish, a specific IgM can be detected in eggs that provides an immune barrier at the surface of the egg as well as protection for the developing fry [15]. In one study on carp, surface IgM^+^ cells were first detected in the head kidneys at 2 weeks post fertilization (wpf) using WCI12 and WCI4 (monoclonal antibodies against the IgM H chain), and carp injected with a T cell-independent antigen (lipopolysaccharide, LPS) developed antibody responses and memory from 4 wpf, while they responded to a T cell-dependent antigen (human gamma globulin, HGG) from 8 wpf [16]. RT-qPCR using primers specific for the IgH constant sequence has been performed to detect the presence of IgH during the early developmental stages of some teleost fish species. In zebrafish, all Ig isotypes effectively responded to LPS challenge from 21 dpf onwards, while IgZ1 responds to LPS challenge faster and more strongly than IgM and IgD at 28 dpf. IgZ-2 transcripts can be detected at 14 dpf [17]. In common carp, whole embryos show constitutive expression of all three Igs (IgM, IgZ1, and IgZ2) as early as 4 dpf with IgM being the predominant form. IgZ1 and IgZ2 expression increases rapidly to peak at 12 dpf whereas IgM peaks at 30 dpf [9]. The appearance of immunoglobulin during the early developmental stage varies considerably among different teleost species due to important differences in developmental status at hatch and the aquatic environment. The presence of Ig molecules in fish embryos and larvae suggests that these molecules are important for defence against pathogens.

The carp is one of the most popular cultured fish in China, and diseases caused by *A. hydrophila* can cause great harm to the carp aquaculture industry. *A. hydrophil*a exhibits antibiotics resistance that is attributed to the indiscriminate use of antibiotics in aquaculture. Vaccines based on antibody-mediated immune responses can enable defence against bacterial infection without the use of antibiotics [18]. In the present study, we cloned a new gene, *Cc*IgZ3 from common carp and detected its expression patterns in adult fish and during embryonic development compare to those of the other known IgHs of this species. The immune responses of *Cc*IgZ3 and IgM after challenge with *A. hydrophil*a by immersion and injection were also compared. Our results provide additional experimental evidence regarding IgZ/T ontogeny and immune function in teleost fish.

## Results

### Molecular cloning and analysis of common carp CcIgZ3

#### Identification of the constant region of CcIgZ3 cDNA in common carp

**Fig. 1:**
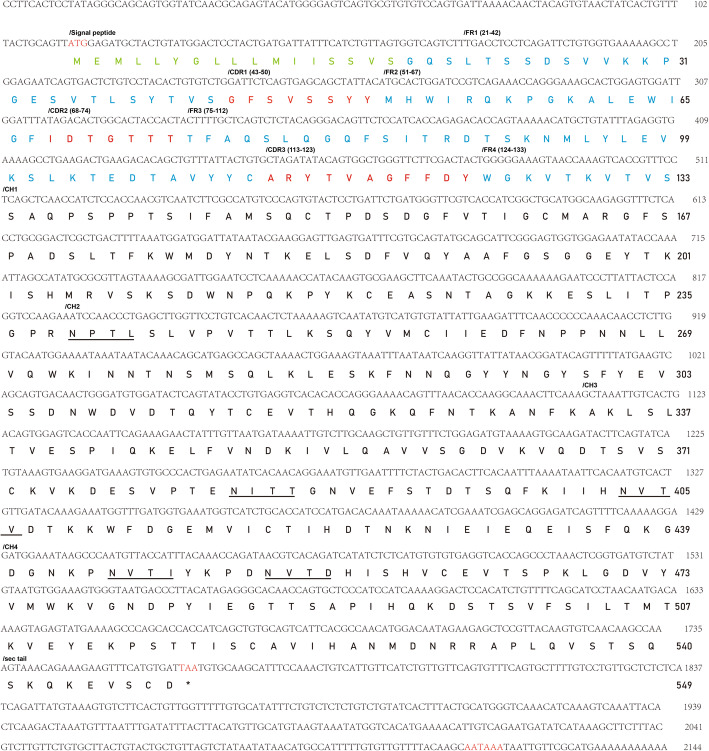
Nucleotide and deduced amino acid sequences for secreted form of common carp (*C. carpio*) IgZ3 (*Cc*IgZ3, GenBank Accession No. MN170744.1). **a** In the nucleotide sequence, the start codon (atg) and the stop codon (taa) are indicated in red. The typical polyadenylation signal (AATAAA) located upstream of the poly(A) tail is indicated in red. **b** In the amino acid sequence, the signal peptide (SP) is marked in green, the framework (FR) is shown in blue and the complementarity determining regions (CDR) are shown in red. **c** The immunoglobulin domains CH1, CH2, CH3, and CH4 and the secreted tail are indicated with slashes above the nucleotide sequence. **d** Potential N-glycosylation sites are underlined

The obtained full-length *Cc*IgZ3 cDNA sequence was 2144 bp with a 5’-UTR of 112 bp, a 3’-UTR of 382 bp and a putative typical polyadenylation signal sequence (AATAAA) located upstream of the poly(A) tail. The deduced *CcIgZ3* amino acid sequence contained 549 aa and spanned the V domain, four constant domains and a secreted tail (Fig. 1). Analysis of the IgT sequence showed the presence of a putative 20 aa signal peptide. IMGT unique numbering was used for the V domain of the *Cc*IgZ3 sequence. The entire C-domain could be divided into four CH domains.

#### Multiple amino acid sequence alignment

**Fig. 2:**
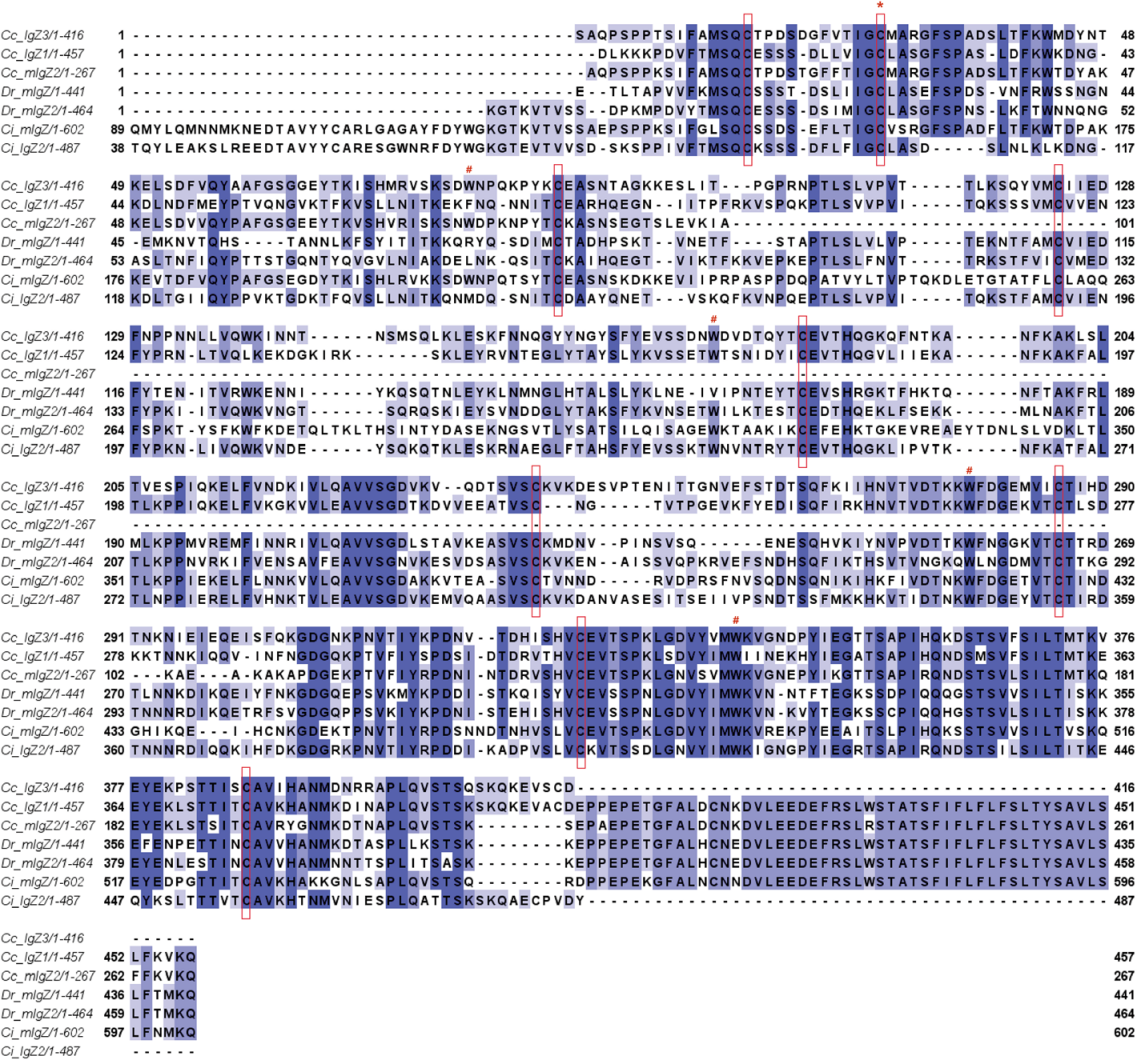
Alignment of the deduced amino acid sequence of *Cc*IgZ3 with homologues from different species. Multiple sequence alignment was performed using Clustal X version 2.1 with the default parameters. The conserved cysteine (C) residues that formed the intrachain disulfide bonds are marked with boxes, and the first conserved C within the CH1 domain for interchain disulfide bond formation is indicated with an asterisk. The conserved tryptophan residues for the folding of the IgSF domain are marked with a hash. The GenBank accession numbers of the IgH sequences used for alignment are Ci_IgZ (ABY76180), Ci_IgZ2 (ABF19723), Dr_IgZ2 (ACH92959), Dr_IgZ (AAT67444), Cc_IgZ1 (BAJ41037), Cc_IgZ2 (BAJ41038)

Alignment of common carp *Cc*IgZ3 with zebrafish IgZ1 and IgZ2, common carp IgZ1 and IgZ2, and grass carp IgZ and IgZ2 showed that *Cc*IgZ3 was composed of four Ig-like constant domains (CH1, CH2, CH3, CH4) and a secretory tail. Conserved cysteine residues for disulfide bond formation and tryptophan residues for folding of the IgSF domain were present in each CH domain of *Cc*IgZ3 (Fig. 2). Five N-linked glycosylation sites were predicted to be present in *Cc*IgZ3 (in CH2, CH3 and CH4) (Fig. 1). The number and distribution of putative N-glycosylation sites, including the sequon NXS or NXT for each domain, varied in different teleost species. The CH1 domain possessed none zero to three putative N-glycosylation sites. There was no N-linked glycosylation site in the *Cc*IgZ3 CH1 domain, unlike in the grass carp sequence CH1 domain(three N-linked glycosylation sites) and the zebrafish sequence CH1 domain (two N-linked glycosylation sites). The first N-linked glycosylation site of *Cc*IgZ3 existed in the CH2 domain and was at the very beginning of the CH2 domain. *Cc*IgZ3 presented two N-linked glycosylation sites in the CH3 and CH4 domains, similar to the grass carp sequence [5].

The percent identity values between the amino acid sequences of all the IgZ subclasses of common carp and those of grass carp, zebrafish and rainbow trout were calculated considering every single domain. The results showed that the identity values among all three IgZ subclasses of common carp ranged from 37.5 to 74.76 %, those between the three IgZ subclasses of common carp and grass carp IgZ1/IgZ2 ranged from 11.59 to 70.21 %, those between the three IgZ subclasses of common carp and zebrafish IgZ1/IgZ2 ranged from 24.18 to 64.13 %, and those between the three IgZ subclasses of common carp and rainbow trout IgT1 ~ IgT3 ranged from 21 to 40.82 %. Interestingly, the identity between the *Cc*IgZ3 CH1 domain and grass carp IgZ CH2 domain (71.26 %) was higher than that between the *Cc*IgZ3 CH1 domain and grass carp IgZ CH1 domain (11.69 %), and the other two IgZ of common carp also showed this characteristic (41.67 % vs. 13.1 in IgZ1, 66.67 % vs. 17.07 % in IgZ2).

#### Phylogenetic analysis

**Fig. 3:**
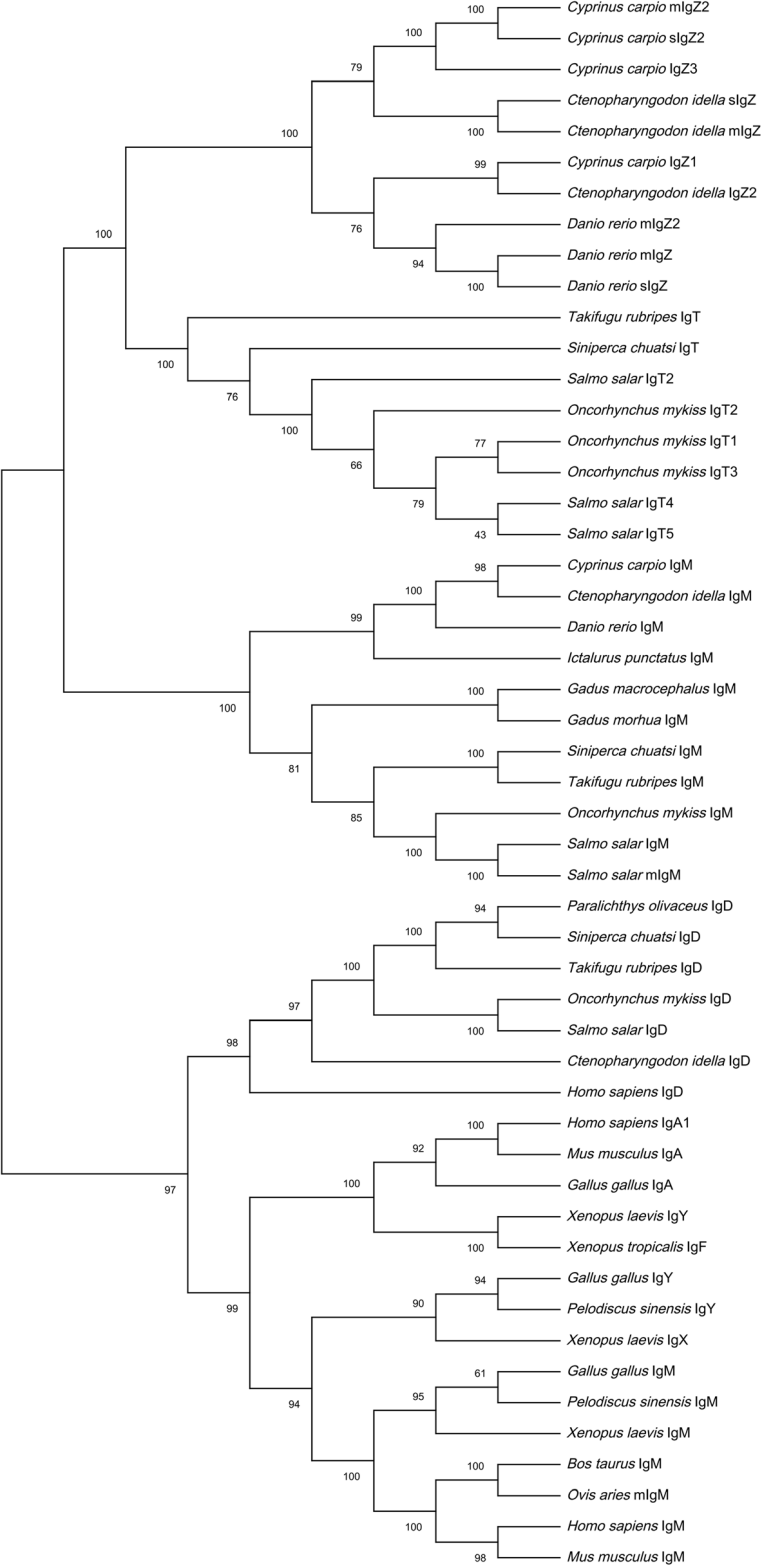
Phylogenetic analysis of vertebrate IgH based on CH sequences. The phylogenetic tree was obtained from a Clustal W alignment and MEGA X by the neighbor-joining method with pairwise gap deletions. The scale indicated the genetic distance. Clusters of teleost Ig sequences are highlighted with different colours. The GenBank accession numbers are listed in Table 2

Phylogenetic analysis was carried out for the deduced amino acid sequences of the constant domains of *Cc*IgZ3 with their counterparts in other vertebrates. The results indicated that *Cc*IgZ3 was grouped with common carp mIgZ2/sIgZ2 and grass carp mIgZ/sIgZ. The IgT/IgZ genes from teleost species formed a distinct cluster separate from those of other IgM and IgD genes identified from fish and other vertebrates (Fig. 3).

### Comparison of the basal expression of CcIgZ3 with that of other Ig molecules in common carp

**Fig. 4:**
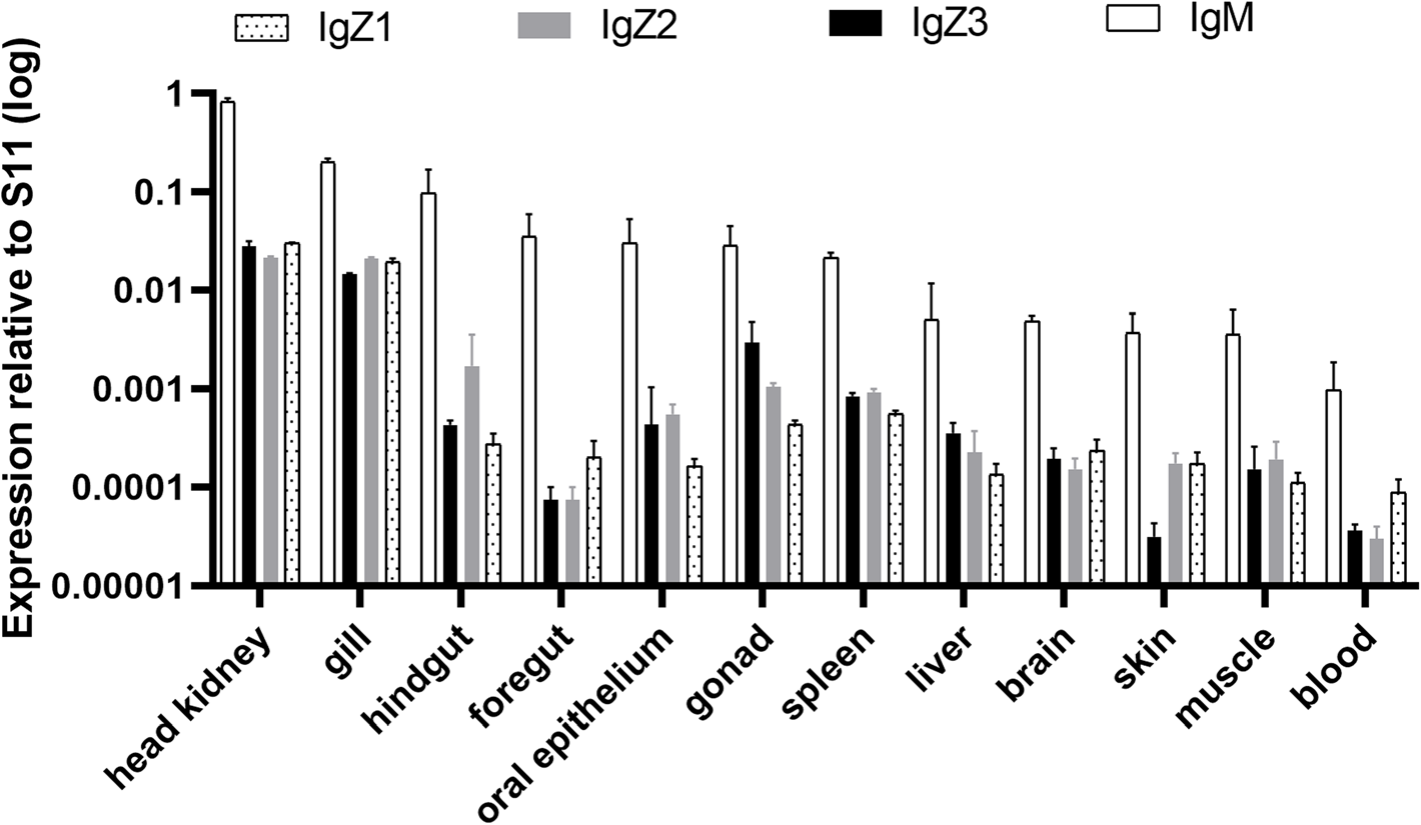
Basal gene expression of Igs in adult common carp. Expression of IgZ1, IgZ2, *Cc*IgZ3 and IgM in head kidney, gill, hindgut, foregut, oral epithelium, gonad, spleen, liver brain, skin, and muscle tissues and in peripheral blood cells. Gene expression was measured by RT-qPCR and normalized to the gene expression of S11. The averages and standard deviations of *n* = 3 samples are plotted

**Fig. 5:**
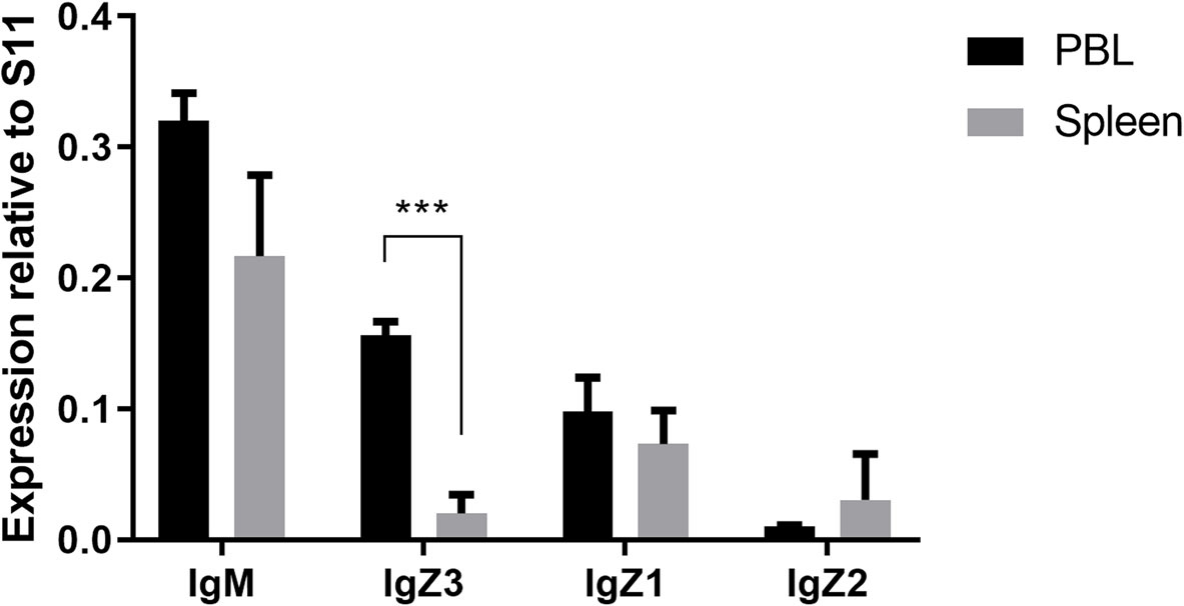
Constitutive Ig transcription levels in leukocytes of common carp. IgZ1, IgZ2, *Cc*IgZ3 and IgM mRNA levels were measured by RT-qPCR in Histopaque-1077 isolated lymphocytes from different tissues (spleens and peripheral blood cells) obtained from naive adult common carp. The results are shown as the mean gene expression relative to the expression of the internal control S11 + standard deviation. The averages and standard deviation of *n* = 3 samples are plotted

To investigate tissue-specific expression patterns, we performed RT-qPCR analysis using gene-specific primers for all three IgZ and IgM genes of common carp in normal adult tissues, including the liver, spleen, head kidneys, gills, skin, gonads, brain, muscle, blood, foregut, hindgut and oral epithelium. *Cc*IgZ3, IgZ1, IgZ2 and IgM were expressed in all the tested tissues, and IgM was the most abundant Ig in all tissues. The expression of *Cc*IgZ3 was found to be highest in the head kidneys, gills, and gonads, followed by the spleen, hindgut, oral epithelium, liver, brain, muscle, foregut, and blood; *Cc*IgZ3 was expressed at a very low level in the skin. IgZ1 and IgZ2 demonstrated expression patterns similar to that of *Cc*IgZ3 in most of the detected tissues except that the lowest transcript levels of IgZ1 and IgZ2 were found in blood (Fig. 4). To compare the expression of the four IgH transcripts in leukocytes, we performed RT-qPCR analysis using gene-specific primers for all three IgZ subclasses and IgM of common carp on isolated leukocytes from peripheral blood cells and spleen tissues. The results showed that a significant difference in *Cc*IgZ3 expression existed between peripheral blood lymphocytes (PBLs) and leukocytes from the spleen. In contrast, no significant differences in IgM, IgZ1 and IgZ2 expression existed between PBLs and leukocytes of the spleen (Fig. 5).

### Comparison of the basal expression of CcIgZ3 with that of other Ig molecules in common carp during different developmental stages

**Fig. 6:**
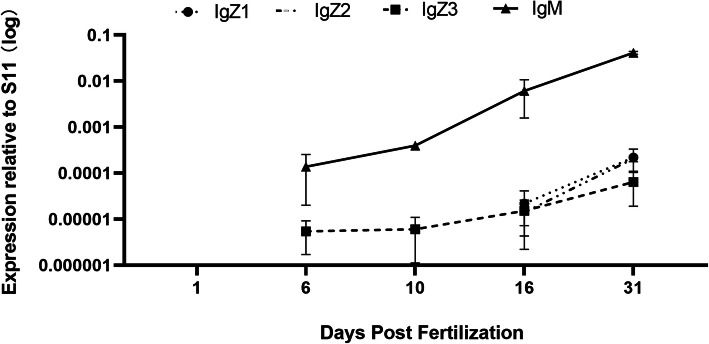
Ontogeny of Igs in common carp as determined by gene expression studies in whole fish. Relative expression of Igs from 1 to 31 dpf. Gene expression was measured by RT-qPCR and S11 was amplified as an internal control. The averages and standard deviations of *n* = 5 samples are plotted. RNA was pooled from 1 to 10 whole fish

**Fig. 7:**
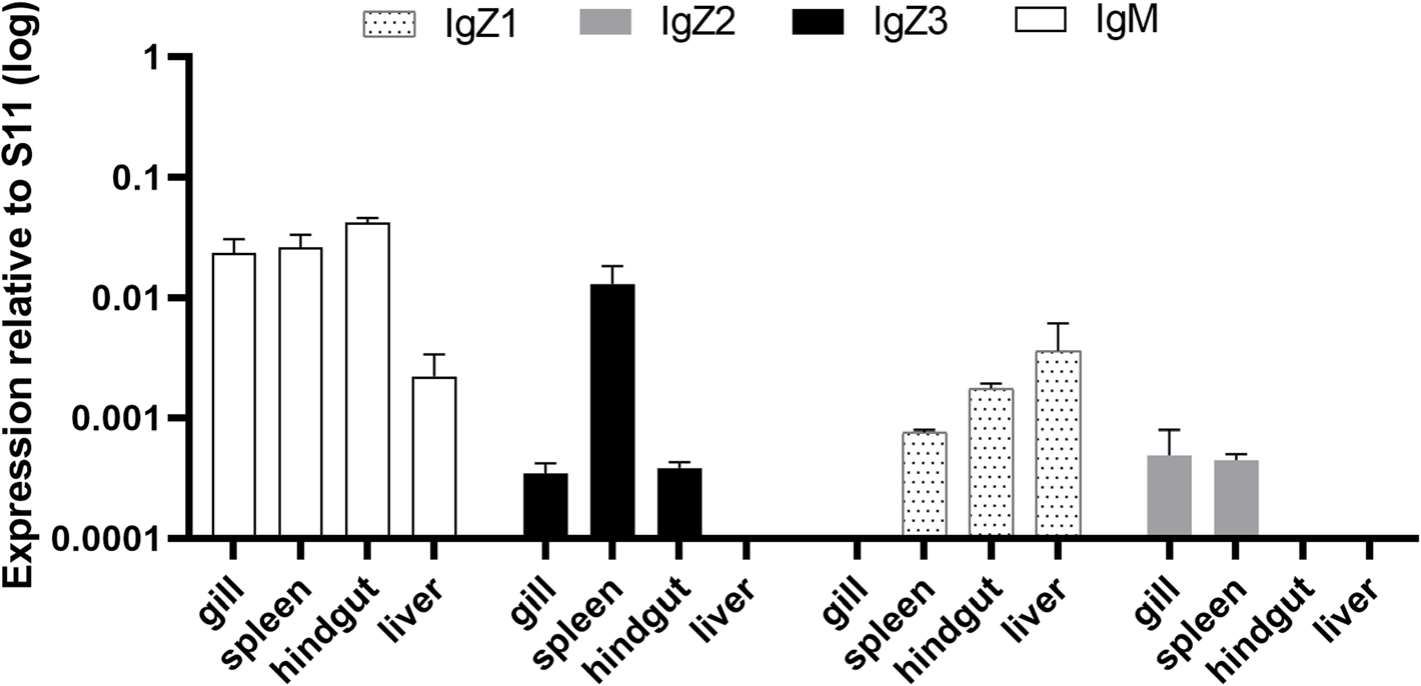
Expression of Igs in specific organs of common carp at the developmental stage of 65 dpf. Antibody expression in gill, spleen, hindgut and liver tissues at 65 dpf. Gene expression was measured by RT-qPCR and normalized to the gene expression of S11. The averages and standard deviations of *n* = 5 samples are plotted

Constitutive expression of the four Ig genes during the early developmental stages of common carp (from 1 to 65 days post fertilization, dpf) was assessed by RT-qPCR (Figs. 6 and 7). Expression of *Cc*IgZ3 and the other three Ig genes was detected beginning at 6 dpf. IgM was expressed at the highest level and was the dominant Ig isotype during the early developmental stages tested (Fig. 6). To investigate the tissue expression patterns of IgH transcripts, the expression levels of *Cc*IgZ3, IgM, IgZ1, and IgZ2 were evaluated in gill, spleen, hindgut and liver tissues from common carp at 65 dpf. The expression of IgM was still higher than that of the other three subclasses, and IgM was the only isotype detectable in all the tested tissues. The genes *Cc*IgZ3, IgZ1 and IgZ2 exhibited varied tissue expression patterns. *Cc*IgZ3 was expressed in the spleen, hindgut and gills but not in the liver, IgZ1 expression was elevated in the liver but undetectable in the gills. IgZ2 expression was detected primarily in the gills and spleen but was hardly detected in the liver and hindgut (Fig. 7).

### Tissue localization of CcIgZ3 and IgM mRNA-expressing cells in common carp at early developmental stages

**Fig. 8:**
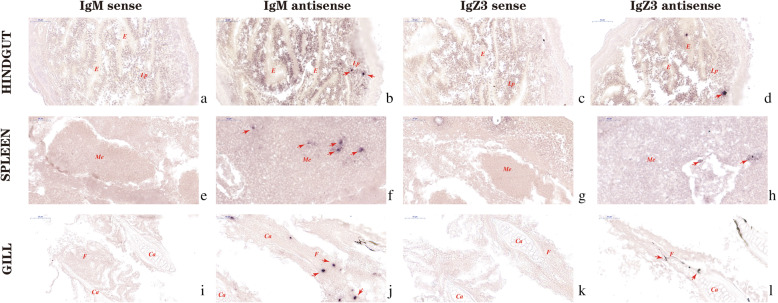
*Cc*IgZ3 and IgM transcripts are differentially expressed in the spleens, gills and hindguts of developing common carp (65 dpf). Sections of spleens, gills and hindguts from developing fish (65 dpf) were hybridized with IgM sense (**a, e, i**) or antisense RNA probes (**b, f, j**), or with, *Cc*IgZ3 sense (**c, g, k**) or antisense RNA probes (**d, h, l**). Lp: laminae propria, E: epithelium, Me: medulla, Ca: cartilaginous cells, F: gill filament. scale bars represent 50 μm

*In situ* hybridization (ISH) analysis of spleen, gills and hindgut sections showed the presence of IgM-expressing cells and *Cc*IgZ3-expressing cells in common carp at the early developmental stage (65 dpf). IgM-expressing cells were detected in both the spleen and gills with strong positive signals. In the spleen, IgM-positive cells were scattered throughout the haematopoietic tissues and in clusters close to the splenic sinus (Fig. 8f). In the gills, IgM-expressing cells were apparently distributed along gill filaments (Fig. 8j). In the hindgut, IgM-expressing cells were detected in the lamina propria, but no positive cells were found in the lamina muscularis (Fig. 8b). No signals were revealed using an IgM mRNA sense probe (Fig. 8a, e, i). *Cc*IgZ3-expressing cells were detected in the spleen, gills and hindgut with comparatively weak positive signals. In the spleen, some single positive cells were found close to the splenic sinus (Fig. 8h). In the gills, CcIgZ3-expressing cells were also detected along gill filaments (Fig. 8l). In the hindgut, CcIgZ3-expressing cells were detected in the lamina propria and epithelium (Fig. 8d). The use of CcIgZ3 mRNA sense probes did not result in any staining (Fig. 8c, g, k).

### Organ‐specific CcIgZ3 and IgM expression after A. hydrophila challenge by different routes

**Fig. 9:**
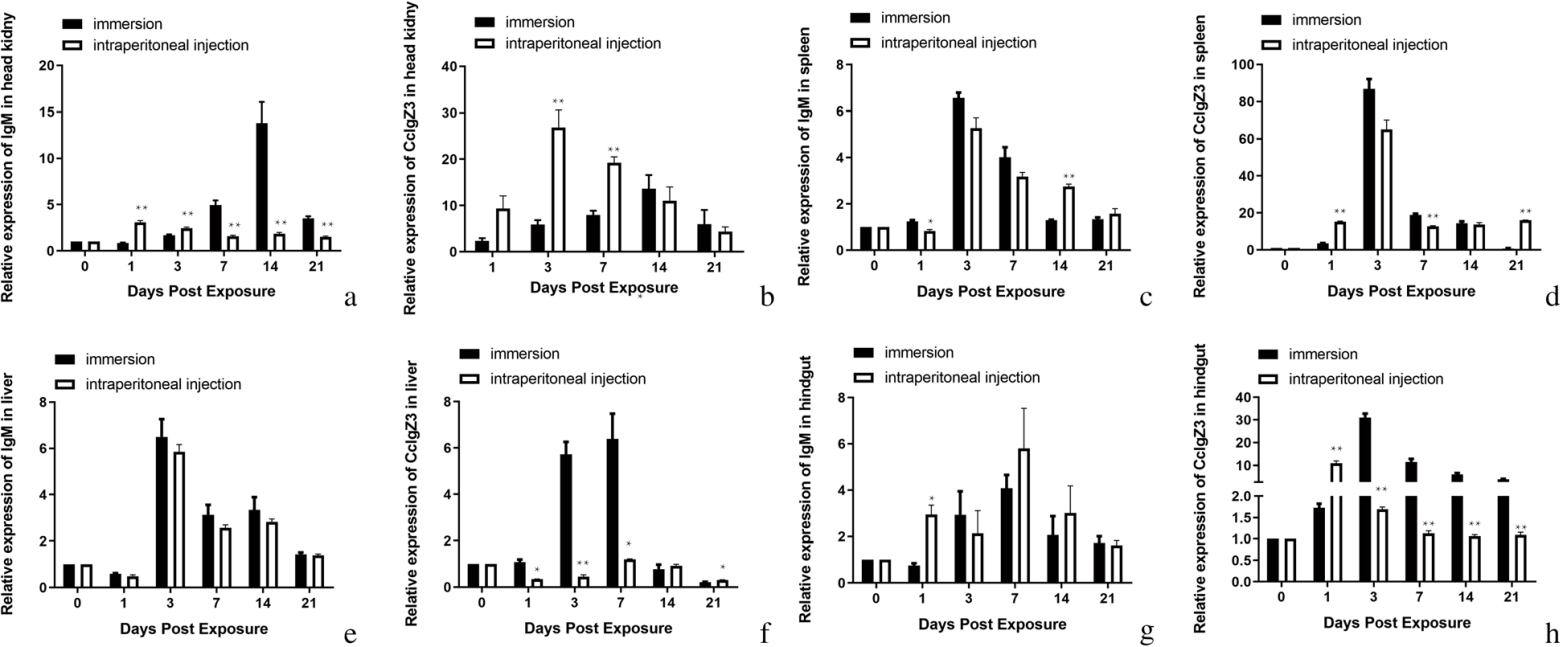
Expression analyses of the Ig genes ***Cc***IgZ3 and IgM genes in different organs of common carp challenged with *A. hydrophila* via i.p. injection or immersion. Time-dependent expression patterns of IgM in the head kidneys (**a**), spleen (**c**), liver (**e**), hindgut (**g**) and of *Cc*IgZ3 in the head kidneys (**b**), spleen (**d**), liver (**f**), hindgut (**h**) after challenge via i.p. injection and immersion. The relative expression levels were detected by RT-qPCR and normalized to the expression at day 0. S11 was used as an internal control. The values represent the means ± standard deviations for each individual fish (*n* = 3). The asterisks indicate significant differences between the i.p. injection group and the immersion group at each time point after the challenge, and the *p* values were calculated by Student’s *t*-test (*, *p* < 0.05)

The RT-qPCR results showed that the mRNA expression of both *Cc*IgZ3 and IgM was upregulated in all tested tissues in the injection and immersion groups compared to the blank control group from 3 days to 21 days, except for *Cc*IgZ3 in the liver in the injection group (0.46-fold at 3 days) (Fig. 9f). Compared with those in the control group, the peak values of *Cc*IgZ3 expression in the immersion group were 86.78-fold in the spleen (Fig. 9d), 31-fold in hindgut (Fig. 9h), 13.61-fold in head kidney (Fig. 9b), and 6.39-fold in the liver (Fig. 9f), while the peak values of CcIgZ3 in the injection group were 65.13-fold in the spleen (Fig. 9D), 10.96-fold in the hindgut (Fig. 9h), 26.91-fold in the head kidneys (Fig. 9b), and 1.21-fold in the liver (Fig. 9f). Compared to those in the injection group, the peak expression levels of *Cc*IgZ3 in the immersion group were higher in the liver (5.28-fold), hindgut (2.83-fold), and spleen (1.33-fold) but lower in the head kidneys (0.51-fold). However, the peak IgM expression was 13.79-fold in the head kidneys (Fig. 9a), 0.57-fold in the spleen (Fig. 9c), 6.5-fold in the liver (Fig. 9e) and 4.07-fold in hindgut (Fig. 9g) in the immersion group compared with the control group; the peak IgM expression was 3.09-fold in the head kidneys (Fig. 9a), 5.27-fold in the spleen (Fig. 9c), 5.85-fold in the liver (Fig. 9e), and 5.8-fold in the hindgut (Fig. 9g) in the injection group compared with the control group. The peak expression of IgM was higher in the head kidneys (4.46-fold), spleen (1.25-fold), and liver (1.11-fold) but lower in the hindgut (0.7-fold) in the immersion group compared with the injection group. The results displayed different tissue expression patterns and smaller fold changes for IgM than for *Cc*IgZ3.

Peak *Cc*IgZ3 and IgM expression occurred at 3–7 days in the spleen, liver, and hindgut in both the immersion group and injection group. However, peak IgM expression in the head kidneys occurred much later in the immersion group (14 days) than in the injection group (1 day). Peak *Cc*IgZ3 expression in the head kidneys also occurred later in the immersion group (14 days) than in the injection group (3 days). In the hindgut, peak *Cc*IgZ3 expression appeared later in the immersion group (3 days) than in the injection group (1 day). In these tissues, the immersion challenge resulted in a slower immune response mediated by *Cc*IgZ3 and IgM than the injection challenge (Fig. 9).

Regarding the response in the hindgut, the upregulation of *Cc*IgZ3 expression was significantly stronger in the immersion group than in the injection group from 3 days to 21 days post challenge, while the expression of IgM was not significantly different during this time period. Compared with that in the control group, *Cc*IgZ3 expression in the hindgut in the immersion group was 1.73-fold at 1 day, 31-fold at 3 days, 11.53-fold at 7 days, 5.97-fold at 14 days, and 3.76-fold at 21 days, while *Cc*IgZ3 expression in the hindgut in the injection group, was 10.96-fold at 1 day, 1.69-fold at 3 days, 1.13-fold at 7 days, 1.06-fold at 14 days, and 1.09-fold at 21 days. Compared with that in the injection group, the expression of *Cc*IgZ3 in the immersion group was 0.15-fold at 1 day, 18.34-fold at 3 days, 10.2-fold at 7 days, 15.63-fold at 14 days, and 3.45-fold at 21 days (Fig. 9h). Compared with that in the control group, the value of IgM expression in the hindgut in the immersion group was 0.75-fold at 1 day, 2.93-fold at 3 days, 4.07-fold at 7 days, 2.07-fold at 14 days, and 1.73-fold at 21 days, while the value of IgM expression in the hindgut in the injection group was 2.94-fold at 1 day, 2.13-fold at 3 days, 5.80-fold at 7 days, 3-fold at 14 days, and 1.62-fold at 21 days. The IgM expression in the immersion group was 0.26-fold that in the injection group at 1 day, 1.38-fold that in the injection group at 3 days, 0.7-fold that in the injection group at 7 days, 0.69-fold that in the injection group at 14 days, and 1.07-fold that in the injection group at 21 days (Fig. 9g). The results showed that *A. hydrophila* challenge, especially by the immersion route, triggered a stronger *Cc*IgZ3 immune response than IgM in the hindgut,

## Discussion

In teleosts, a new Ig isotype apart from IgM and IgD, IgZ/T, has been discovered, and more than one subclass of IgZ/T has been found in some fish species. Although alignment of sequences in these subclasses has indicated that the member of each IgZ/IgT subclass exhibit some common structural features, specific gene organization and conserved amino acid residues, their expression and distribution patterns in immune organs and tissues clearly vary, implying that there are functional differences among these IgZ/T molecules [19, 20].

To date, in common carp, two IgZ subclasses have been reported, IgZ1 and chimeric IgZ2, both of which have membrane-bound and secretory forms. In the current study, alignment of *Cc*IgZ3 with other IgZ sequences revealed the presence of conserved cysteine residues that participate in the formation of intrachain disulfide bonds in each CH domain of all three IgZ subclasses in common carp. In addition, the conserved cysteine residues for interchain disulfide bonds existed in all three IgZ CH1 domains. However, the conserved tryptophan residues for the folding of the IgSF domain were found only in the CH domains of *Cc*IgZ3 and IgZ2, and the positions of the tryptophan residues in the CH4 domains of *Cc*IgZ3 and IgZ2 in the common carp were different from those in rainbow trout and zebrafish. The sequence of the secretory tail was conserved among the three subclasses. Alignment of the CH sequences indicated that the CH4 domain was the most highly conserved domain and that the CH1 domain exhibited a lower level of sequence identity. Phylogenetic analysis showed that *Cc*IgZ3 was grouped with IgZ2, and the sequence identities for the two molecular CH1 and CH4 domains were 69.52 and 74.49 %, respectively. In addition, the *Cc*IgZ3 CH1 domain was highly similar to the grass carp IgZ CH2 domain, while the CH1 domain sequence identity was very low (11.69 %). These differences made it possible to discriminate the IgZ subclasses on the basis of their expression and localization.

In the current study, the results of constitutive expression analyses of different subclasses in normal tissues showed that the IgZ2 chimera was expressed at slightly higher levels in mucosal tissues, including the gills, skin, hindgut and oral epithelium, than IgZ1 and *Cc*IgZ3; however, IgZ2 was expressed at a lower level in the blood than the other three subclasses, which may indicate that the IgZ2 chimera is preferentially expressed at mucosal sites. This result corroborates previous findings indicating that IgZ2 is expressed at higher levels in the gut and gills than in other tissues [9]. Unlike all three subclasses of IgZ, IgM was predominantly expressed in all organs, including both lymphoid and nonlymphoid organs and tissues. IgZ1 was expressed systemically, as indicated by the finding that it was expressed at higher levels than IgZ2 and *Cc*IgZ3 in blood. As the third member of the group of new Ig subclasses, *Cc*IgZ3 was expressed at higher levels in the gonads and liver than IgZ1 and IgZ2. To investigate whether *Cc*IgZ3 has immune function, an *A. hydrophila* challenge experiment was performed. We found that *Cc*IgZ3 expression was strongly induced in the hindgut during *A. hydrophila* challenge by immersion, although basal *Cc*IgZ3 expression was not very high in the hindgut. The teleost gut interacts with foreign pathogens from the water directly and is a main site for pathogen entry in teleost fish. Teleost Igs elicit the mucosal immune response in GALT via specific B lymphocyte proliferation and local antibody secretion within mucosa-associated lymphoid tissues (MALTs) [21, 22]. The posterior segment of the teleost intestine has been discovered to contain several immune cell types and has been shown to play a more important role in immune responses to pathogen invasion than the first segment and the second segment of the teleost intestine [13, 22]. The *Cc*IgZ3 immune response in the hindgut of common carp indicates that this molecule may play an important role in protecting the host from *A. hydrophila* infection, and the *Cc*IgZ3 immune response is even stronger than that of IgM.

Ontogeny studies have been conducted on Igs in several fish species, including IgZ1 and IgZ2 in common carp. In previous studies, IgM and IgZ have been found to be expressed during the same stage of development in fugu [11]and trout [23], but IgZ was the first detected antibody in zebrafish [5], In common carp, IgZ1 and IgZ2 can be detected as early as 4 dpf, and tissue-specific expression is first observed in the head and trunk kidneys, which exhibit the highest expression [9, 10]. To compare the different subclasses, we first detected IgM, *Cc*IgZ3, IgZ1, and IgZ2 expression during the early developmental stage from 1 to 31 dpf by RT-qPCR. In the current study, IgM and *Cc*IgZ3 were expressed at all time points beginning at the same stage(6 dpf) and their expression increased gradually. However, IgZ1 and IgZ2 were expressed beginning at 16 dpf, and their expression levels at a greater rate than those of *Cc*IgZ3 to exceed those of CcIgZ3 at 31 dpf. In addition, analysis of tissue-specific expression at 65 dpf showed that IgM was the only detectable antibody in the four tissues and it was comparatively abundant in the spleen, hindgut and gills. *Cc*IgZ3 existed primarily in the spleen, followed by the gills and hindgut. The IgZ1 gene was expressed at relatively higher levels in the liver, and then in the hindgut and spleen, and the IgZ2 gene was expressed in the gills and spleen but not in the hindgut and liver. These observations may indicate the existence of functional differences among these subclasses. Furthermore, the production of these three subclasses of IgZ might be attributable to the differentiation and maturation of B lymphocytes residing within systemic lymphoid tissues and MALTs. The subpopulations of IgZ-positive B lymphocytes need further investigation.

Although considerable IgZ/T characterization has been conducted, little information is available on the *in situ* detection of IgZ-expressing cells in fish, especially during development [11, 24, 25]. Based on the results of RT-qPCR analysis of Ig expression during development, ISH was employed to investigate the *Cc*IgZ3-expressing cells and IgM-expressing cells in the tissues of carp at the developmental stage of 65 dpf. The ISH results clearly showed that the localization of *Cc*IgZ3-expressing cells in the spleen, gills and hindgut was similar to that of IgM-expressing cells; however, the transcripts levels of the two antibodies were different, as their positive reactivity varied. Our findings imply that ISH might reveal the distribution and localization of *Cc*IgZ3-expressing cells even in the early developmental stage in common carp, and our results might help to clarify the production of *Cc*IgZ3 and the maturation of *Cc*IgZ3-positive B lymphocytes.

## Conclusions

In conclusion, a third IgH member in common carp, *Cc*IgZ3, was cloned and characterized and the expression and localization of *Cc*IgZ3 compared with those of IgM, IgZ1 and IgZ2 during larval development were investigated. *Cc*IgZ3 and IgM in systemic immune tissues and GALTs of common carp challenged with *A. hydrophila* via injection and immersion were compared. We hope that our findings help to expand the information on adaptive immunity in common carp and other teleost fish. Further research is required to understand the roles of the different IgZ/T subclasses and their corresponding B lymphocytes.

## Methods

### Ethics statement

 All experiments on live animals were carried out in accordance with relevant guidelines and regulations. The protocol was approved by the Animal Experimental Ethics Committee of Shandong Normal University (Permit Number: AEECSDNU2017004). All efforts were made to minimize suffering. The study was carried out in compliance with the Animal Research: Reporting of In Vivo Experiments (ARRIVE) guidelines.

### Experimental animals

Common carp (*C. carpio*) weighing between 150 and 200 g were obtained from the Fresh Water Fishery Research Institute of Shandong Province (China) and maintained in tanks with aerated freshwater at 22–25℃. The fish were acclimated to the aquaria for at least 2 weeks before being used in experiments. The fish were anaesthetized by immersion in a solution of tricaine methanesulfonate (MS222, Sigma-Aldrich) and killed. Blood, head kidney, spleen, liver, gill, foregut, midgut, hindgut, oral epithelium, skin, gonad, and muscle tissue samples were collected immediately, frozen in liquid nitrogen, and stored at -80℃ until use [26, 27]. All animal experiments were approved by the Committee on the Ethics of Animal Experiments of Shandong Normal University.

### Molecular cloning and analysis of common carp CcIgZ3

#### Total RNA extraction and first‐strand cDNA synthesis

Each frozen sample was ground in a mortar with liquid nitrogen, and then total RNA was isolated using the TRIzol universal reagent (Tiangen, China). The quality and concentration of all total RNA samples were assessed using a NanoDrop Spectrophotometer (Thermo Scientific, USA). First-strand cDNA was synthesized from 2 µg total RNA with a FastQuant RT Kit (with gDNase) (Tiangen, China) according to the manufacture’s instructions [28, 29]. Total RNA from the collected samples was extracted following the procedure above, and then the cDNA was stored at -80 ℃ until use for real-time quantitative PCR (RT-qPCR).

### Molecular cloning and sequencing of common carp CcIgZ3

A common carp *CcIgZ3* cDNA fragment was first amplified by PCR with primers IgZ3 F1 and IgZ3 R1, which were designed based on known IgZ sequences from teleost fish (GenBank Accession No.: *D. rerio* AY643750, EU732710.1, AY643750, *C.idella* DQ478943, GQ201421, *C. carpio* AB004105, AB598367, AB598368, AB598369). cDNA from common carp head kidneys was used as the template. The reactions steps were as follows: 3 min of initial denaturation at 94℃; 35 cycles of 1 min of denaturation at 94℃, 30 s of annealing at 55℃, and 1 min of extension at 68℃; and 5 min of final extension at 68℃. Ex Taq HS (Takara) was used for PCR, and the PCR products were loaded on a 1 % agarose gel and visualized by staining the gel in 0.1 mg/mL-ethidium bromide. The DNA amplified in each reaction system by PCR was purified using a Gel Extraction Kit (Tiangen), inserted into the pMD19-T vector (Takara) and transformed into competent TOP 10 *E. coli* cells for sequencing.

Subsequently, rapid amplification of cDNA ends (RACE) was performed using a 3’-Full RACE Core Set (Takara) and a SMARTer RACE cDNA Amplification Kit (Takara) to obtain the full-length *Cc*IgZ3 cDNA sequences with specific primers that were designed based on the obtained partial sequence. The 3’-Full RACE Core Set (Takara) was utilized to obtain 3’-unknown regions with the specific forward primers and the adaptor primers listed in Table 1. The first round of PCR was performed using the primer pair IgZ3 race-3’outer primer/3’RACE outer primer, under the following conditions: one cycle of 94℃ for 3 min; 30 cycles of 94℃ for 30 s, 57℃ for 30 s, and 72℃ for 2 min; and a final extension step at of 72℃ for 10 min. The resultant product was diluted and reamplified in a second round of PCR using the primer pair IgZ3 race-3’inner primer/3’RACE inner primer under the same reaction conditions. All PCR products were purified using a Gel Extraction Kit (Tiangen) and then were cloned into the pMD19-T vector (Takara) for sequencing. For the 5’ RACE reaction, first strand cDNA was synthesized from 1 µg of total spleen lymphocyte RNA after adding the 5’ RACE adapter to RNA following the manufacturer’s instructions. PCR was performed with the 5’ RACE Ready cDNA samples using an Advantage 2 PCR Kit (Clontech) according to the manufacturer’s specifications and the specific primers shown in Table 1. The reaction steps were as follows: one cycle of 95℃ for 1 min, 30 cycles of 95℃ for 30 s and 68℃ for 3 min, and a final extension step at 68℃ for 3 min. All PCR products were purified using a NucleoSpin Gel and PCR Clean-Up Kit (Takara) and then cloned into the pRACE vector (Takara) for sequencing.

**Table 1:** List of primer sequences used in the study

Name	Sequence(5’-3’)	Application
IgZ3-F1	TCAGCTCAACCATCTCCACC	ccIgZ3 gene cloning
IgZ3-R1	GCTTGTTGACACTTGTAACGGAG	ccIgZ3 gene cloning
IgZ3race-3’ outer primer	CCCTTATTACTCCAGGTCCAAGAAA	ccIgZ3 race gene specific primer
IgZ3race-3’ inner primer	ATTACTCCAGGTCCAAGAAATCCAA	ccIgZ3 race gene specific primer
IgZ3race-5’ outer primer	GGATGGGAGCACTGGTTGTGCCCTCT	ccIgZ3 race gene specific primer
IgZ3-1817F2	AGGGCAGCAGTGGTATCAAC	ccIgZ3 gene cloning
IgZ3-1817R2	GCAACAGGACAAAAGCACTGA	ccIgZ3 gene cloning
IgZ3-1803F3	CAACGCAGAGTACATGGGGA	ccIgZ3 gene cloning
IgZ3-1803R3	GAGCAACAGGACAAAAGCACT	ccIgZ3 gene cloning
S11qF	CCGTGGGTGACATCGTTACA	gene expression analysis
S11qR	TCAGGACATTGAACCTCACTGTCT	gene expression analysis
IgZ3-qF3	TGCGGACTCGCTGACTTTTA	gene expression analysis
IgZ3-qR3	GACAGGAACCAAGCTCAGGG	gene expression analysis
IgM-qF1	GGTGTTTGTGTCTTGGCTTGCT	gene expression analysis
IgM-qR1	CGTCCACTTGGAATCATTAACTG	gene expression analysis
IgZ1-qF	GAGAATTTCTACCCCAGG	gene expression analysis (reference)
IgZ1-qR	GACCTTCAGTATTCACTCTG	gene expression analysis(reference)
IgZ2-qF1	GCTGAAGCTAAAGCTAAAGCTCC	gene expression analysis (reference)
IgZ2-qR1	TGAGAGACCCGATCTGTGTTAAT	gene expression analysis(reference)
IgZ2-qF2	AATTCTGAAGCACCTCACTAGA	gene expression analysis(reference)
IgZ2-qR2	CACACACATGAGAGACCCGAT	gene expression analysis(reference)
IgZ3_situ-F4	TTCACGCCAACATGGACAATAGAA	ccIgZ3 *in situ* hybridization
IgZ3_situ-R4	TGTTTGACCCATGCAGTAAAGTG	ccIgZ3 *in situ* hybridization
IgM_situ-F2	ATGACCCTGACGTGTTATGTGAA	IgM *in situ* hybridization
IgM_situ-R2	CTCAAAGAAGCAAGAAGCCACAA	IgM *in situ* hybridization

### Bioinformatic analysis of common carp CcIgZ3

The full-length sequence of *Cc*IgZ3 was confirmed by PCR using sequence-specific primers IgZ3-1817F2/R2 and IgZ3-1817F3/R3. The open reading frames (ORFs) and deduced protein sequences of *Cc*IgZ3 were predicted using the ORF Finder program and by blasting genomic stretches against protein databases at NCBI (blastx) [30]. The locations of Ig domains were predicted using the InterProScan program, the PROSITE Database and the NCBI Conserved Domain Databases. Posttranslational modifications were predicted with the NetNGlyc 1.0 program. The theoretical isoelectric point and the molecular weight of the amino acid sequence were calculated using the ExPASy Compute pI/Mw program. Multiple sequence alignment was conducted using Clustal X version 2.1 with the default parameters, and the resulting alignment was adjusted manually [12]. Based on the alignment, a phylogenetic tree was generated from the deduced amino acid sequence using the neighbour-joining method with MEGA X. All the sequences used for the phylogenetic analysis are listed in Table 2.

**Table 2:** Sequences of Igs used for phylogenetic tree construction and multiple sequence alignment

Protein	species	*Accession* *number*	Protein	species	*Accession* *number*
chicken IgA	*Gallus gallus*	AAB22614.2	human IgE	*Homo sapiens*	AAB59424
chicken IgM	*Gallus gallus*	CAA25762.1	human IgG1	*Homo sapiens*	CAA75032
chicken IgY	*Gallus gallus*	CAA30161.1	human IgM	*Homo sapiens*	CAB37838
pacific cod	*Gadus macrocephalus*	AKL81191	human IgM	*Homo sapiens*	CAC20458
atlantic cod	*Gadus morhua*	CAA41680	channel catfish IgD	*Ictalurus punctatus*	AAC60133
Chinese soft-shelled turtle	*Pelodiscus sinensis IgM*	ACU45376	channel catfish IgD	*Ictalurus punctatus*	ADF56020
Chinese soft-shelled turtle	*Pelodiscus sinensis IgY*	ACU45374	channel catfish IgH	*Ictalurus punctatus*	AAA79003
mandarin fish IgD	*Siniperca chuatsi*	ACO88906	channel catfish IgM	*Ictalurus punctatus*	A45804
mandarin fish IgM	*Siniperca chuatsi*	AAQ14862	little skate IgM	*Leucoraja erinacea*	AAB04671.1
mandarin fish IgT	*Siniperca chuatsi*	AAY42141	little skate IgW	*Leucoraja erinacea*	AAA49546
cattle IgM	*Bos taurus*	AAC71048	mouse IgA	*Mus musculus*	AAH10324
common carp IgM	*cyprinus carpio*	BAA34718	mouse IgG	*Mus musculus*	AAB59658
common carp IgZ1	*cyprinus carpio*	BAJ41037	mouse IgM	*Mus musculus*	AAB59650
common carp mIgZ2	*cyprinus carpio*	BAJ41038	rainbow trout IgD	*Oncorhynchus mykiss*	AAW66976
common carp sIgZ2	*cyprinus carpio*	BAJ41039	rainbow trout IgM	*Oncorhynchus mykiss*	AAB27359
common carp IgZ3	*cyprinus carpio*	MN170744	rainbow trout IgM	*Oncorhynchus mykiss*	AAW66972
zebrafish IgM	Danio rerio	AAK96442	rainbow trout IgT1	*Oncorhynchus mykiss*	AAW66978
zebrafish IgM	Danio rerio	AAT67447	rainbow trout IgT2	*Oncorhynchus mykiss*	AAV48553
zebrafish mIgZ	Danio rerio	AAT67444	human IgA1	Homo sapiens	BAC87456.1
zebrafish mIgZ2	Danio rerio	ACH92959	human IgD	Homo sapiens	EAW81936
zebrafish sIgZ	Danio rerio	AAT67446	sheep mIgM	Ovis aries	AAA51379
Fugu rubripes IgM	*Takifugu rubripes*	BAD26619	African clawed frog IgM	Xenopus laevis	AAA49774
Fugu rubripes IgT	*Takifugu rubripes*	BAD69712	African clawed frog IgX	Xenopus laevis	CAA32027
Fugu rubripes IgD	*Takifugu rubripes*	BAD34541	Atlantic salmon IgM	*Salmo salar*	AAB24064
Fugu rubripes IgH	*Takifugu rubripes*	BAD89297	Atlantic salmon IgT2	*Salmo salar*	ADD59873
Atlantic salmon IgD	*Salmo salar*	ADD59896	Atlantic salmon mIgM	*Salmo salar*	ACN10415
grass carp IgD	*Ctenopharyngodon idella*	ADK66818	African clawed frog IgY	*Xenopus laevis*	CAA33212
grass carp IgM	*Ctenopharyngodon idella*	ABD76396	tropical clawed frog IgF	*Xenopus tropicalis*	AAH87793
grass carp sIgZ	*Ctenopharyngodon idella*	ADD82655	rainbow trout IgT3	*Oncorhynchus mykiss*	ANW11927
grass carp IgZ2	*Ctenopharyngodon idella*	ABF19723	Japanese flounder IgD	*Paralichthys olivaceus*	BAB41204.1

### Gene expression studies of common carp igs

#### Basal expression of ig isotypes in organs of common carp

For tissue expression analysis, total RNA was isolated from the head kidneys, spleens, livers, blood, skin, gills, foreguts, midguts, hindguts, oral epithelia, gonads and brains of normal common carp and then reverse-transcribed into first-strand cDNA as described above. PCR was conducted with specific primers as indicated in Table 1. with SuperReal PreMix Plus (SYBR Green, Tiangen, China). The RT-qPCR amplification program for S11 (as a standard) consisted of 30 cycles of 94℃ for 30 s, 55℃ for 30 s, and 72℃ for 30 s using the primers S11F/S11R, and the amplification programs for IgM, IgZ1, IgZ2, and *Cc*IgZ3 consisted of 30 cycles of 94℃ for 30 s, 55℃ for 30 s, and 72℃ for 30 s using specific primers (listed in Table 1). The reactions for the standards and target genes were conducted in parallel tubes. The relative expression of each Ig gene was calculated and normalized to the expression of S11.

#### Basal expression of ig isotypes in leukocytes isolated from different tissues of adult common carp

Whole blood was collected from the caudal vein for isolation of PBLs (peripheral blood lymphocytes) with a heparinized syringe and centrifuged at 4℃ and 500×g for 10 min, the serum was then removed. The cells were diluted 6-fold in the same volume of RPMI-1640 medium as the original volume of blood at room temperature and then placed on ice. The spleen was dissected from each anaesthetized fish and placed in a sterile plastic culture dish containing 5 mL of RPMI-1640 with 100 U/mL penicillin G and 100 mg/mL streptomycin (Sigma-Aldrich, USA). A single-cell suspensions was first obtained from the spleen by teasing apart the tissue with sterile dissecting scissors, repeatedly aspirating it and then passing it through a 100 μm nylon mesh with RPMI-1640 medium. A total volume of 10mL of single-cell suspension was gradually layered upon the same volume of Histopaque 1077 (Sigma-Aldrich, USA) in a 50 mL centrifuge tube, and the tube was centrifuged 500×g for 40 min at 4℃. Leukocytes were collected from the interface layer and washed three times with medium [31, 32]. The cell quantity and viability were determined with 0.4 % trypan blue (Sigma-Aldrich, USA), and cells were collected for RT-qPCR analysis [33]. The expression of Ig genes was calculated and normalized to that of S11.

### Relative expression of ig isotypes during the development of common carp

For expression analysis of Igs during different developmental stages, fertilized eggs (n = 100) were obtained, and total RNA was extracted from embryos or larvae 1, 6,10,16 or 31 dpf using a FastQuant RT Kit (with gDNase) (Tiangen) following the steps described above. Ig expression in tissues from common carp at 65 dpf, including gill, spleen, hindgut and liver tissues was detected by RT-qPCR following the same procedure.

### ISH

#### Synthesis of RNA probes

Common carp IgM and *Cc*IgZ3 cDNAs sequences were amplified with primers (Table 1.) and subcloned into the pSPT18 vector (Roche). The primers IgZ3_situ_F4 and IgZ3_situ_R4 were designed to amplify a 244 bp product corresponding to the constant region of the *Cc*IgZ3 CH4 domain. The primers IgM_situ_F2 and IgM_situ_R2 were designed to amplify a 435 bp product corresponding to the constant region of common carp IgM CH4 domain. The cycling protocol was as follows: denaturation at 94℃ for 3 min; 35 cycles of 94 ℃ for 30 s, 58℃ for 30 s and 72 ℃ for 30 s, and final extension step at 72 ℃ for 10 min. The PCR products were visualized on 1 % agarose gels containing ethidium bromide. The fragments were purified using a TIANgel Midi Purification Kit (Tiangen), inserted into the pSPT18 vector (Roche) and transfected into competent *E. coli* DH5α cells. Plasmid DNA from four clones was purified and sequenced. Sequence analysis was performed to confirm the sequence identity and insert orientation.

To generate RNA probes, the clones were digested with EcoR-I or Hind-III, and the fragments were purified on an agarose gel and used for *in vitro* transcription reactions with a DIG RNA Labelling Kit (Roche). Transcription was performed with SP6 RNA polymerase and T7 RNA polymerase according to the protocol to generate antisense and sense RNA probes.

#### ISH

Spleen, gill and hindgut tissues were aseptically extracted from fish and fixed in 4 % paraformaldehyde in phosphate-buffered saline (PBS)-H_2_O_DEPC_ for at least 4 h. The tissues were then immersed in a 15 % sucrose solution for 8 h and transferred to a 30 % sucrose solution for soaking overnight. Tissue embedded in optimum cutting temperature (OCT) compound was sectioned at a thickness of 4 μm and mounted onto poly-L-lysine-coated slides. The frozen slides were removed from the freezer, fixed in paraformaldehyde (4 % in PBS, pH 7.4) for 20 min, washed three times with DEPC-treated buffer (pH 7.4) and permeabilized with protein K (5 µg/mL) buffer at 37℃ with gentle rocking. After washing in PBS-glycine buffer, the sections were washed twice with PBS-H_2_O_DEPC_. Prehybridization was performed by incubating the sections with prehybridization buffer (Servicebio) for 60 min at 37℃. DIG-labelled antisense RNA probes (1 µg/ml) were applied with hybridization solution to the tissues, and the tissues were incubated at 55℃ overnight in a moistened chamber. To remove the hybridization solution, the sections were washed sequentially with 5× SSC, 1×SSC, 0.5×SSC and 20 % formamide (50 min). The tissue sections were blocked with 5 % serum blocking reagent for 30 min at room temperature. The anti-DIG-AP antibody used for detection was diluted 1:500 in blocking buffer solution containing 5 % serum. The sections were washed twice with PBS; subsequently, BCIP/NBT reagent (Roche) was applied according to the protocol. The reaction was visualized and documented using a bright-field microscope.

### Organ‐specific IgM and CcIgZ3 expression after A. hydrophila challenge through different routes

Fifty common carp were divided into two groups for immune stimulation and challenged with *A. hydrophila via* intraperitoneal (i.p.) injection or immersion as previously described [28]. Briefly, the *A. hydrophila* used in the study was obtained from the China Center for Type Culture Collection and incubated in LB medium at 28℃ overnight under continuous shaking. For the injection challenge, *A. hydrophila* was inactivated in 0.5 % formalin at 4℃ overnight and then suspended in sterile 0.1 M PBS. Each fish was challenged by i.p. injection with 5 × 10^7^ CFU per fish. For the immersion challenge, cultured *A. hydrophila* was added to the aquarium to a concentration of 1 × 10^8^ CFU/ml. After being treated for 40 min, the carp were removed and transferred to a tank containing fresh water. On days 0, 1, 7, 14, 21 and 28 after stimulation, three fish from each group were anaesthetized with MS-222. Tissue samples, including spleen and hindgut samples, were taken, frozen in liquid nitrogen, and used for total RNA extraction and subsequent RT-qPCR analysis following the same procedure described above. The relative mRNA expression was determined via relative quantification with the comparative cycle threshold (Ct) (2^(−ΔΔCt)^) method; the level of target mRNA was normalized with respect to S11, an internal reference gene, and the results are expressed relative to the levels in the unchallenged control fish (denoted as day 0) [33].

## Data Availability

The dataset supporting the conclusions of this article is available in the GenBank (https://www.ncbi.nlm.nih.gov/nuccore/1806102469) and the accession number is MN170744.1.

## Abbreviations

Ig: Immunoglobulin
IgH: Immunoglobulin heavy chain
ISH: *In situ* hybridization
MALT: Mucosa-associated lymphoid tissues
GALT: Gut-associated lymphoid tissue
wpf: Weeks post fertilization
dpf: Days post fertilization
